# Proximity Ligation *In situ* Assay is a Powerful Tool to Monitor Specific ATG Protein Interactions following Autophagy Induction

**DOI:** 10.1371/journal.pone.0128701

**Published:** 2015-06-02

**Authors:** Thierry Gauthier, Aurore Claude-Taupin, Régis Delage-Mourroux, Michaël Boyer-Guittaut, Eric Hervouet

**Affiliations:** Université de Franche-Comté, Laboratoire de Biochimie, EA3922 « Estrogènes, Expression Génique et Pathologies du Système Nerveux Central », SFR IBCT FED4234, UFR Sciences et Techniques, France; Niigata University School of Medicine, JAPAN

## Abstract

Macroautophagy is a highly regulated intracellular degradation process which has been extensively studied over the last decade. This pathway has been initially described as a non selective process inducing the degradation of parts of the cytoplasm as well as organelles at random. Nevertheless, over the last few years, new research highlighted the existence of a more selective autophagy pathway specifically recruiting some organelles or aggregates to the autophagosomes in order to induce their degradation. These selective autophagy pathways such as aggrephagy, mitophagy, pexophagy or xenophagy, involve the intervention of a cargo, the material to be degraded, cargo adapters, the molecules allowing the recruitment of the cargo to the autophagosome, and the proteins of the ATG8 family which link the cargo adapters to the autophagosome. One of the main questions which now remain is to develop new techniques and protocols able to discriminate between these different types of induced autophagy. In our work, we studied the possibility to use the P-LISA technique, which has been recently developed to study endogenous *in vivo* protein interactions, as a new technique to characterize the ATG proteins specifically involved in bulk or selective autophagy. In this manuscript, we indeed demonstrate that this technique allows the study of endogenous ATG protein interactions in cells following autophagy induction, but more interestingly that this technique might be used to characterize the ATG proteins involved in selective autophagy.

## Introduction

Macroautophagy (hereafter called autophagy) is a catabolic process that leads to the identification, transport and the degradation of cytosolic constituents to the lysosome. More than 40 ATG proteins are related to the initiation, elongation and maturation of a double membrane vesicle, referred as autophagosome, during autophagy. One family has been described to be particularly important for vesicle formation in yeast as well as mammals, the ATG8 family. In mammals, these homologues of the only yeast ATG8 are divided in two subfamilies: the LC3 family (LC3A, LC3B, LC3C) required in the early phases of autophagosome formation and the GABARAP family (GABARAP, GEC1/GABARAPL1 (GL1), GATE-16/GABARAPL2) which seems to be more involved in the elongation and closure of autophagosomes.[1] The ATG8 members are synthetized as cytosolic pro-proteins and cleaved by ATG4 enzymes at a C-terminal Glycine to give the mature form of these proteins (form I) before their conjugation onto phospholipids to give the lipidated form of ATG8s (form II). Starvation or hypoxic stress have been described to induce autophagy in a non selective manner and induce the degradation and/or recycling of damaged cellular components in order to regulate cellular homeostasis. More recently, a selective autophagy leading to the specific degradation of intracellular components (ubiquitinylated proteins, damaged mitochondria, endoplasmic reticulum, peroxysomes, ribosomes) has been described. This selective process requires the interaction of specific protein cargo adapters with the ATG8 proteins, linked to the membrane of the autophagosome as an anchor point to recruit the cargo into the autophagosome. These cargo adapters, such as SQSTM1/P62, NBR1, NIX/BNIP3L or NDP52/CALCOCO2, all interact with LC3-II *via* a LIR domain (LC3 interacting domain) and with their ligand to be degraded, the cargo using an UBA domain (ubiquitin associated).[2] NIX/BNIP3L is required for the selective degradation of mitochondria, called mitophagy, while NBR1 is indispensable for the selective degradation of peroxisomes, called pexophagy.[3] NDP52 regulates the selective degradation of DICER and AGO2 and thus regulates miRNA activity [4] whereas SQSTM1/P62 is implicated in the selective degradation of ubiquitinylated proteins.[5] These different autophagy cargo adapters can then interact with different ATG8 proteins through an AIM WXXL-like motif (ATG8-family interacting motif).[6] This high number of putative interactions between cargo adapters and ATG8s might explain the existence of numerous types of selective autophagy in the cells. This hypothesis has been confirmed by a recent *in vitro* study performed by Berhends and collaborators in order to characterize the cellular autophagy network which revealed a complex network of interactions between autophagy proteins.[7]

Autophagy deregulation has been associated with numerous human pathological disorders even if the mechanism still remains unknown. For example, inactivation or modulation of the expression of several autophagy genes have been reported in cancer cells. Indeed, *BECLIN-1*, *BIF-1* and *UVRAG*, three essential autophagy genes have been classified as tumor suppressor genes and are frequently inactivated in cancer cells leading to the promotion of cell proliferation and aggressiveness.[8,9,10] Defects and mutations in autophagy genes have also been frequently observed in neurodegenerative disorders such as Alzheimer’s disease, or familial Parkinson’s disease.[11] Since then, the development of techniques to efficiently monitor autophagy levels in cell and tissue models became a challenge to better characterize autophagy protein expression, function and deregulation in these pathologies. This information will be essential in the future to characterize autophagy levels or autophagy gene and protein expressions as potential diagnostic markers or therapeutic targets.

Several techniques currently used to quantify autophagy levels in cells and tissues are based on the detection of proteins associated to the autophagosomes. Therefore, the ATG8 proteins, and in particular LC3, are the preferential targets for autophagy level quantification. SQSTM1/P62 has also been extensively studied by the autophagy community since this protein was defined as a specific substrate of autophagy.[12] Nevertheless, more recent studies demonstrated that the levels of SQSTM1/P62 are highly regulated by different cellular stresses and cannot be considered anymore as exclusively correlated to autophagy levels. Therefore, western blotting experiments are used to quantify both the LC3-II and LC3-I forms and the amounts of SQSTM1/P62 protein, and *in situ* analysis of GFP-LC3 vesicles in transfected cells are frequently performed. An improved version of the latter approach has been obtained by the expression of a double fusion protein mRFP-GFP-LC3B which allows the identification and quantification of both autophagosomes (in yellow) and lysosomes (in red). Indeed, it has been described a loss of GFP fluorescence, but not red fluorescence, in acidic compartments (*e*.*g*. lysosomes).[13] In spite of their highly informative power on autophagosome formation, these techniques are not adapted to study bulk versus selective autophagy. Regarding selective autophagy, researchers concentrate on the study of localization and quantification of cargo adapters such as GFP-P62 or GFP-NBR1.[14] However, due to high levels of aggregation following protein overexpression,[15] variable levels of expression of exogenous GFP or the difficulty to accurately quantify the number of GFP vesicles in transfected cells, in particular when willing to perform co-localization quantification, these models remain difficult and problematic to use. Here, we propose the use of a recently new described technique, called proximity ligation *in situ* assay (P-LISA) (S1 Fig), to monitor endogenous ATG8/cargo adapters interactions to accurately analyze and quantify non selective and selective autophagy in cells.

In this publication, we demonstrate that SQSTM1/LC3B, SQSTM1/GL1, NIX/LC3B and NIX/GL1 interactions can be quantified using P-LISA and are effectively correlated to autophagy levels as shown by the comparison with former techniques. Moreover, our work shows that SQSTM1/LC3B interaction is increased following different autophagy inducers whereas NIX/LC3B and NIX/GL1 interactions are mainly regulated by mitochondria stressors. Altogether, our data describe for the first time, the use of P-LISA in order to easily and efficiently discriminate between different types of autophagy targeting endogenous non overexpressed autophagy proteins.

## Material and Methods

### Antibodies and reagents

The following antibodies were used: monoclonal anti-P62/SQSTM1 (Santa Cruz, sc-28359; dilutions: IF/P-LISA 1/200, WB 1/1000), polyclonal anti-LC3B (Sigma, L8918; dilutions: IF/P-LISA 1/100, WB 1/3000), polyclonal anti-GABARAPL1 (Proteintech, 11010-AP, dilution: IF/P-LISA 1/100), monoclonal anti-NIX (Santa Cruz, sc-166332; dilution: IF/P-LISA 1/100), polyclonal anti-rabbit-HRP conjugate (P.A.R.I.S, BI2407; dilution: 1/10 000), polyclonal anti-mouse-HRP conjugate (P.A.R.I.S.; dilution: 1/10 000), polyclonal anti-rabbit-Alexa 488 (Life Technologies, BI2413C; dilution 1/1 000), and polyclonal anti-mouse-Alexa 555 (Life Technlogies; dilution: 1/1000). Cell culture reagents were purchased from Invitrogen. EBSS (E3024), BafA1 (B1793), Rapa (R8781) and CCCP (C2759) were purchased from Sigma-Aldrich. Rot (AC13237) was purchased from Acros organics.

### Cell culture

MDA-MB-436 and MCF-7 cells were obtained from ATCC (HTB-130 and HTB-22). Cells were cultured at 37°C with 5% CO_2_ atmosphere in DMEM 1g/L glucose (Dominique Dutscher, L0066) supplemented with 5% SVF (Dominique Dutscher, S1810), 1% penicillin/streptomycin (Dominique Dutscher, L0018) and 0.1% Fungizone (PAA, P11-001). MCF-7 control and MCF-7 overexpressing FLAG-GABARAPL1-6His were available in the laboratory and cultured as described above with the exception of 10% SVF.[16]

### Plasmids and transfection

The pGFP-LC3 vector was kindly provided by Dr. Elazar (The Weizmann Institute of Science, Rehovot, Israel). The pEGFP-NIX was kindly provided by Dr. Xiao-Ming Yin[17] and the pGFP-mRFP-LC3B (ptf-LC3) vector was purchased from Addgene (21074). Transient transfections were performed in 24-well plates using 0.5 μg of DNA and 1μL of JetPrime reagent (Polyplus Transfection, 114–07) according to manufacturer’s protocol.

### Western blotting

Cells were scraped, harvested and lysed in RIPA buffer (50 mM Tris-HCl, pH 8, 150 mM NaCl, 1% Triton X 100, 0.5% DOCA, 0.1% SDS) supplemented with 0.1% protease inhibitors (104 mM AEBSF, 1.5 mM pepstatin A, 1.4 mM E-64, 4 mM bestatin, 2 mM leupeptin, 80 μM aprotinin). Lysates were sonicated ten times for 5 sec. 40μg of protein were loaded and separated on a 12.5% sodium dodecyl sulfate-polyacrylamide gel electrophoresis (SDS-PAGE) before being transferred onto a polyvinylidenedifluoride (PVDF) membrane (Bio-Rad, 162–0177). Membrane was blocked with 5% nonfat milk in Tris-buffered saline supplemented with Tween 20 (TBS-T) (20 mM Tris-HCl, pH 7.6, 137 mM NaCl, 0.1% Tween 20) and incubated with primary antibodies overnight at 4°C under gentle agitation. Immunoreactive bands were detected using goat horseradish peroxidase (HRP)-coupled secondary (anti-mouse or anti-rabbit antibodies) and the *p*-coumaric acid-enhanced chemiluminescent (PCA-ECL) solution. Signals were acquired using the ChemiDocXRS+ (Biorad, France) and quantified using the Biorad Image Lab software (version 4.0) (Biorad, France).

### Immunofluorescence and Proximity Ligation in situ Assay (P-LISA)

Cells were cultured for 24 h on coverslips and then fixed with 4% paraformaldehyde pH 7.4 in PBS (137 mM NaCl, 2.7 mM KCl, 10 mM Na_2_HPO_4_, 2 mM KH_2_PO_4_) during 15 min at room temperature. Permeabilization was performed in cold methanol for 20 min at 4°C.

For immunofluorescence, blocking was realized with 0.1% tween-TBS with 5% BSA for 1h at 37°C. Incubations with primary antibodies were performed overnight at 4°C, and then cells were rinsed 3 times with 0.1% tween-TBS. Incubations with secondary antibodies were performed for 1 h at 37°C and then cells were rinsed 3 times with 0.1% tween-TBS, stained with DAPI (4',6'-diamidino-2-phénylindole) and mounted using Vectashield Hardset mounting medium (Vector Laboratories, H-1400).

For P-LISA, all incubations were performed in a humidity chamber and according the OlinkBioscience’s recommendations using Duolink In Situ Detection Reagents Red kit (DUO92008, Sigma-Aldrich, France). Briefly, coverslips were blocked with Blocking solution (82007, Olinkbioscience) for 45 min at 37°C and then incubated with primary antibodies previously diluted in Antibody diluent (82008, Olinkbioscience) overnight at 4°C. Coverslips were washed 3 times for 5 min in T-PBS buffer under gentle shaking and then incubated with PLA probes MINUS and PLUS corresponding to the primary antibodies using Duolink In Situ PLA Probe Anti-Goat MINUS (DUO92006, Sigma-Aldrich, France), Duolink In Situ PLA Probe Anti-Mouse PLUS (DUO92001, Sigma-Aldrich, France), Duolink In Situ PLA Probe Anti-Mouse MINUS (DUO92004, Sigma-Aldrich, France) and Duolink In Situ PLA Probe Anti-Rabbit MINUS (DUO92005, Sigma-Aldrich, France) for 2 h at 37°C. Then, coverslips were washed 3 times for 5 min in T-PBS buffer under gentle shaking and then incubated with a DNA ligase previously diluted in Ligation buffer for 30 min at 37°C. Coverslips were washed 3 times for 5 min in T-PBS buffer under gentle shaking and incubated with a DNA polymerase previously diluted in Amplification buffer for 90 min at 37°C. Finaly, coverslips were rinsed for 10 min in presence of DAPI under gentle shaking and then washed 2 min with (0.02X, 0.2X, or 2X (30 mM sodium citrate; 300 mM sodium chloride) SCC buffer, and then 2 min in 70% ethanol. Dried coverslips were mounted with Vectashield Mounting Medium (Vector Laboratories, H-1000). Fluorescence was visualized with an Olympus IX81 confocal microscope (Olympus, France) and pictures acquisition was realized using a DP75 camera.

Finally, immunofluorescence images were analyzed using the “ImageJ” software while P-LISA images were analyzed using the “BlobFinder”software available for download from www.cb.uu.se/~amin/BlobFinder. Interactions were quantified by counting the number of dots per cell as well as the intensity of signal per dot. An increase of intensity is the consequence of a concentration of interactions in the same cellular dots. [18] In the different figures, each bar (Mean ± SEM) represents the mean obtained from the quantification of signals observed in about 200 cells chosen randomly in 5 different fields from 3 independent experiments.

### Statistical Analysis

Differences between means were analyzed using t-test with GraphPad Prism 5 software. Co-localization experiments were analyzed using Pearson and the Image J (plug-in Coloc-2) software. * (p<0.05), ** (p<0.01), *** (p<0.001) and **** (p<0.0001)

## Results

### P-LISA can be used for the detection of autophagy protein interactions in breast cancer cell models

Previous reports described the importance of multiple interactions between autophagy proteins during the course of autophagosome formation.[2,5,6,7,19] Moreover, recent data suggested that specific interactions between members of the ATG8 family (LC3B, GABARAP or GABARAPL1) and cargo adapters, such as SQSTM1/P62 or NIX, were essential to induce the selective degradation of target proteins or organelles during a new processus called selective autophagy. For example, SQSTM1/LC3B interaction has been previously reported in cells overexpressing GFP-LC3B, and the authors demonstrated that this interaction favored the SQSTM1 localization in autophagosomes.[19,20,21] Moreover, additional interactions of SQSTM1 with other ATG8 proteins (LC3A, LC3B, LC3C, GABARAP, GABARAPL1 and GABARAPL2) have been reported in ATG8-overexpressing cell models [7]. Unfortunately, these data were mostly observed in transfected cells and it is known that overexpression of exogenous proteins might lead to non specific interactions or the formation of GFP aggregates as already pointed out.[15] Therefore, we decided to develop a specific P-LISA protocol to quantify *in cellulo* endogenous autophagy protein interactions.

P-LISA is a multi-step technique leading to the detection of specific fluorescent dots linked to close proximity protein interactions (range below 40 nm) (S1 Fig). This technique is based on the use of two different primary antibodies of different species which are specific of two proteins supposed to interact in the cells. The advantage of this technique is the detection of endogenous proteins in the cellular context without the need of overexpression. Moreover, the maximal distance between the two proteins to get a signal should not exceed 40 nm, a distance which is below the sensitivity of confocal microscopy used for co-localization experiments.

**Fig 1:**
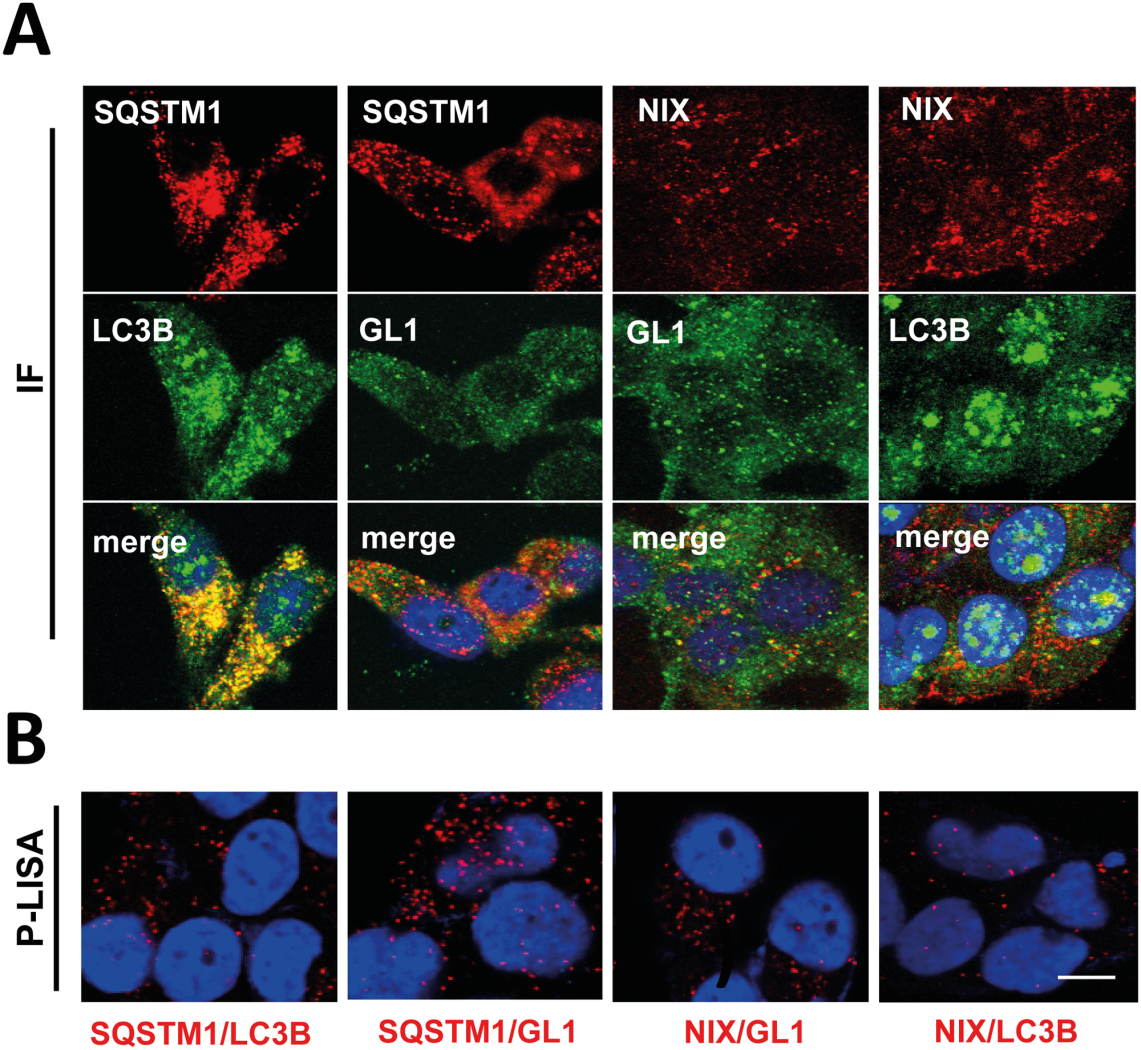
Detection of SQSTM1/LC3B, SQSTM1/GL1, NIX/GL1 and NIX/LC3B interactions by P-LISA. (**A**) MDA-MB-436 cells were cultured for 24 h at 37°C and 5% CO_2_, fixed, permeabilized, blocked with 5% BSA, incubated with rabbit anti-LC3B, rabbit anti-GL1, mouse anti-SQSTM1 or/and mouse anti-NIX antibodies overnight at 4°C and then with an Alexa Fluor 488 goat anti-rabbit and an Alexa Fluor 555 goat anti-mouse, respectively, for 1 h. The cells were then analyzed using a confocal microscope. (**B**) For P-LISA, the protocol was performed according to the manufacturer’s recommendations using the same antibodies as described above. Nuclei were stained with DAPI. Each picture is representative of a typical cell staining observed in 10 fields chosen at random. Scale bars: 20μm.

First, we compared the possibility to use P-LISA in breast cancer cells (MDA-MB-436) to detect the interaction between LC3B (an ATG8 family member) and SQSTM1 (a cargo adapter). Immunofluorescence (IF) experiments confirmed the expression of these proteins and their partial co-localization in the MDA-MB-436 cells (Fig 1A left panel). However, co-localization of proteins detected by IF does not necessarily mean interaction. Moreover, co-localization quantification of ATG proteins in immunostained cells is difficult to analyze due to the large distribution of these proteins in the cell. Since our LC3 antibody seemed to present a rather low specificity and induced the detection of non specific nuclear signals, which has already been described by others,[22] we wondered whether P-LISA would give a more specific result in our model. Therefore, we developed a specific P-LISA protocol to quantify *in cellulo* endogenous SQSTM1 and LC3B interactions. During these P-LISA experiments, using two specific primary antibodies directed against SQSTM1 and LC3B, we detected quantifiable specific P-LISA dots corresponding to the interaction of endogenous LC3B and SQSTM1 in MDA-MB-436 cells without the need to overexpress these proteins (Fig 1B left panel). We then asked whether this protocol could be used for the study of other autophagy-related protein interactions and therefore targeted the interactions between SQSTM1 and GABARAPL1 (GL1, another ATG8 family member) or interactions of a second cargo adapter NIX with LC3B or GL1 in MDA-MB-436 cells (Fig 1). Immunofluorescence experiments confirmed the expression of NIX and LC3 proteins in our models and their partial co-localization in MDA-MB-436 cells (Fig 1A). Then, the use of NIX/GL1 P-LISA revealed the presence of dots (a mean of 11.6 dots/cell) in MDA-MB-436 which quantification was facilitated thanks to the specificity of P-LISA staining. We obtained similar results regarding the interaction between LC3B and NIX (a mean of 18.9 dots/cell) (Fig 1B).

**Fig 2:**
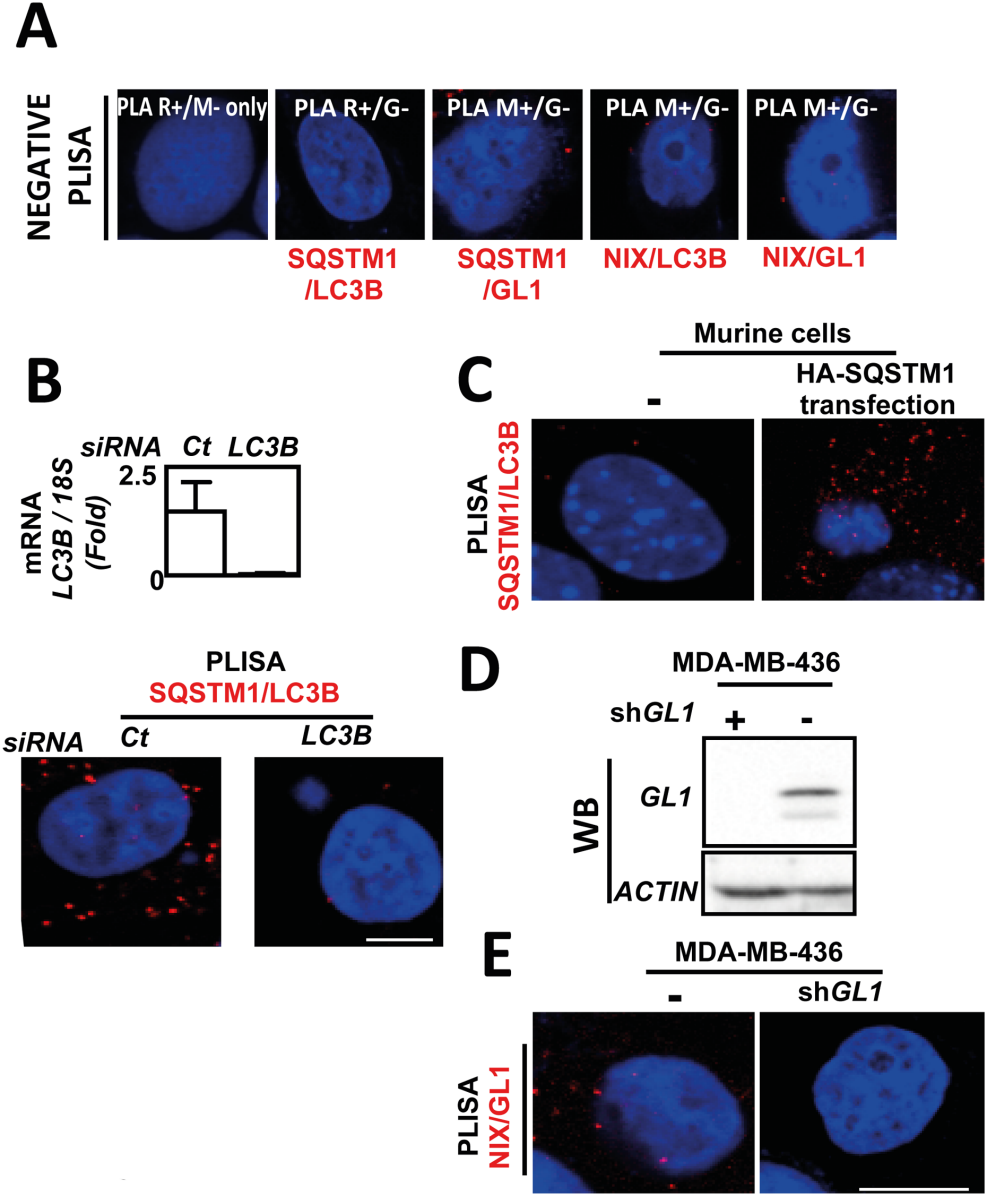
Technical controls demonstrating the specificity of P-LISA signals. (**A**) MDA-MB-436 cells were cultured for 24 h at 37°C and 5% CO_2_. P-LISA were performed according to the manufacturer’s recommendations. No primary antibodies were added before performing P-LISA with PLA R+ (anti-rabbit) and PLA M- (anti-mouse) (left panel); P-LISA SQSTM1/LC3B was also performed with PLA R+ (anti-rabbit) against LC3B and with PLA G- (anti-goat) unable to recognize SQSMT1 (left panel). Similar controls were performed for P-LISA SQSTM1/GL1, P-LISA LC3B/NIX and P-LISA GL1/NIX. (**B**) Quantification of *LC3B* mRNA expression in MDA-MB-436 cells following *LC3B* siRNA transfection analyzed using qRT-PCR (top panel). Quantification of SQSTM1/LC3 interactions detected by P-LISA in MDA-MB-436 cells transfected or not with *LC3B* siRNA (bottom panel) according to the manufacturer’s recommendations using rabbit anti-LC3B and mouse anti-SQSTM1 antibodies. (**C**) Absence of SQSTM1/LC3B P-LISA signals in murine cells since the anti-SQSTM1 antibody is specific of the human SQSTM1 protein and restoration of P-LISA signals when these cells were transfected with a vector coding the human HA-SQSTM1 protein. (**D**) Absence of GL1 protein was validated using western blotting in MDA-MB-436 cells expressing or not a *GABARAPL1* shRNA (**E**) Quantification of NIX/GL1 interactions was performed by P-LISA in MDA-MB-436 cells expressing or not a *GABARAPL1* shRNA using rabbit anti-GL1 and mouse anti-NIX antibodies.

To confirm that these signals observed in MDA-MB-436 cells were indeed specific, we performed several technical controls (Fig 2). First, we performed P-LISA controls without primary antibodies (Fig 2A left panel) or using one secondary PLA probe antibody incompatible with the primary antibodies used. As expected, both controls did not produce any P-LISA signals in the cells (Fig 2A). Second, we used siRNA targeting *LC3B* expression in our models (Fig 2B top panel). We also observed that the specific *LC3B* siRNA also strongly decreased the number of SQSTM1/LC3B P-LISA fluorescent dots, data which demonstrated the specificity of the technique (Fig 2B bottom panel). To confirm these data, we performed a control using murine cells since the murine SQSTM1 protein does not contain the human epitope recognized by the anti-SQSTM1 antibody used in our P-LISA experiments. Once again, we confirmed the specificity of our technique since we observed no SQSTM1/LC3B P-LISA signal in murine cells but showed the restoration of P-LISA signals when these cells were transfected with a vector coding the human HA-SQSTM1 protein (Fig 2C). At last, we performed NIX/GL1 P-LISA in MDA-MB-436 cells stably expressing a shRNA targeting *GABARAPL1*.[16] Absence of GL1 in these cells was confirmed using westen blotting (Fig 2D). Our data confirmed that the cells which did not express GL1 following shRNA expression did not present any P-LISA staining (Fig 2E).

**Fig 3:**
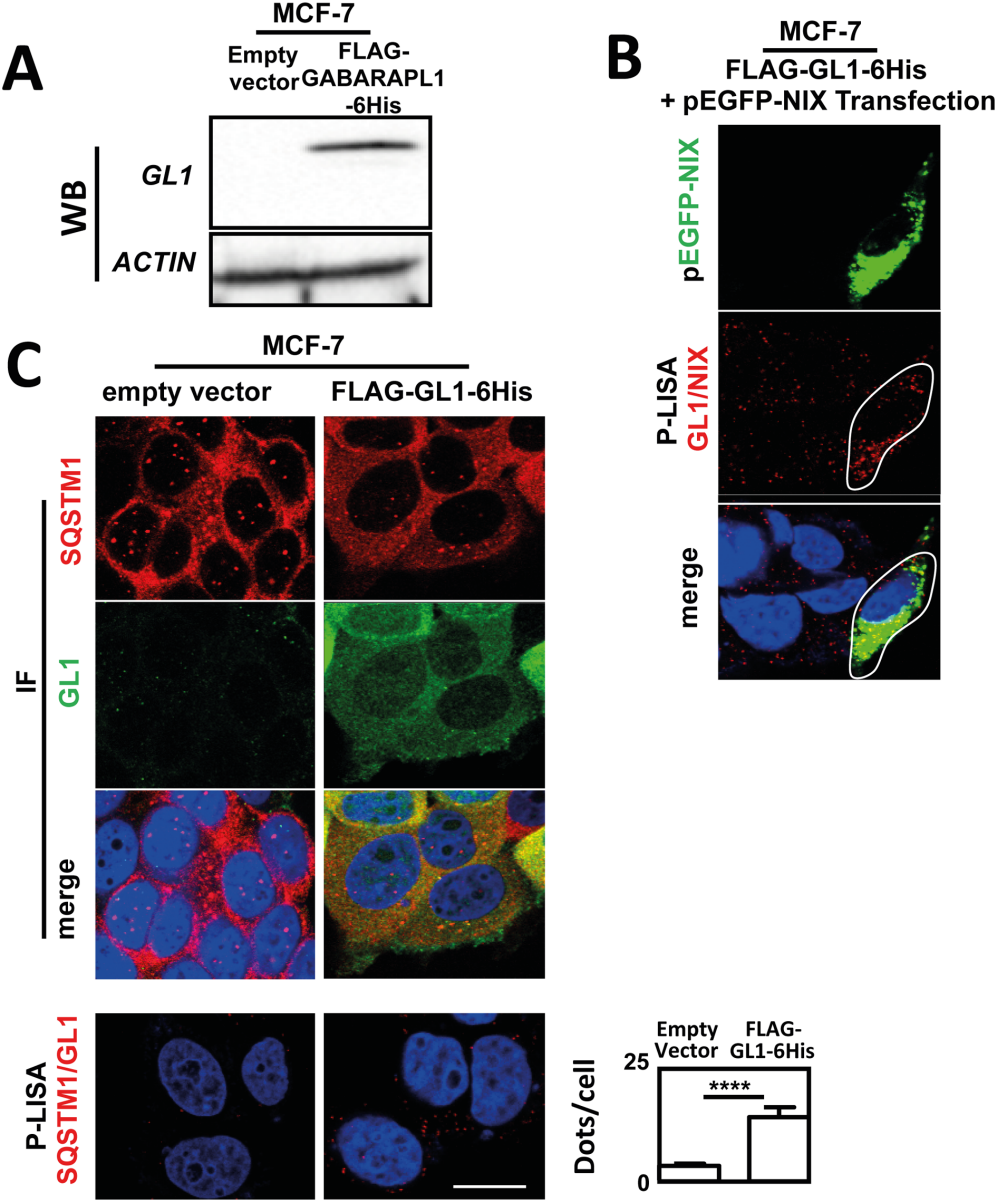
Increase of SQSTM1/GL1 and NIX/GL1 interactions quantified by P-LISA in MCF-7 overexpressing FLAG-GABARAPL1-6His and GFP-NIX. (A) Expression of GL1 protein was analyzed using western blotting in MCF-7 expressing or not FLAG-GL1-6His. (**B**) MCF-7 Control or MCF-7 FLAG-GL1-6His cells were cultured for 24 h at 37°C and 5% CO_2_, fixed, permeabilized, blocked with 5% BSA, incubated with mouse anti-SQSTM1 and rabbit anti-GL1 antibodies antibodies overnight at 4°C and then with an Alexa Fluor 555 goat anti-mouse and an Alexa Fluor 488 goat anti-rabbit, respectively, for 1 h. The cells were then analyzed using a confocal microscope (top panel). For P-LISA, the protocol was performed according to the manufacturer’s recommendations using mouse anti-SQSTM1 and rabbit anti-GL1 antibodies (bottom panel). (**C**) MCF-7 FLAG-GL1-6His cells were cultured for 24 h at 37°C and 5% CO_2_ and transfected with pEGFP-NIX plasmid and then fixed and permeabilized. P-LISA was performed as precognized by the manufacturer using rabbit anti-GL1 and mouse anti-NIX antibodies. Nuclei were stained with DAPI. Each picture is representative of a typical cell staining observed in 10 fields chosen at random. Scale bar: 20μm.

Since a decreased expression of GL1, a member of the ATG8 family, has been frequently observed in breast cancer (BC) cells, we decided to use the MCF-7 cell line, which has been described to present an undetectable expression of GL1 in western blotting and a weak basal signal in IF, to validate the specificity of the SQSTM1/GL1 and NIX/GL1 P-LISA (Fig 3). Western blotting (Fig 3A) and immunofluorescence experiments (Fig 3B) indeed confirmed the low GL1 expression in MCF-7 control cells (stably transfected with an empty vector) and the presence of this protein in MCF-7 cells stably overexpressing FLAG-GL1-6His, as previously described.[16] These data also showed an increase in the co-localization of GL1 and SQSTM1 in overexpressing cells compared to non-expressing cells (Fig 3B top panel). Regarding SQSTM1/GL1 P-LISA staining, as expected, few fluorescent dots were observed in MCF-7 control cells (3.9 dots per cell) while a significant increase of SQSTM1/GL1 P-LISA signals was quantified in MCF-7 stably overexpressing FLAG-GL1-6His (15.4 dots per cell) (p<0.0001, Fig 3B bottom panel). MCF-7 cells stably overexpressing FLAG-GL1-6His cells expressed a very low level of NIX protein associated with a low signal of NIX/GL1 P-LISA signals in these cells (Fig 3C). Nevertheless, the transfection of the pEGFP-NIX vector and the overexpression of GFP-NIX in these cells, increased NIX/GL1 P-LISA signals confirming that these interactions are NIX-dependent (Fig 3C).

Altogether, these data demonstrated the feasibility and the specificity of P-LISA to detect specific autophagy protein interactions in BC cell models. Moreover, we showed that, in our models, the quantification of P-LISA signals is easier and more accurate than the quantification of co-localization signals in IF experiments.

### Quantification of SQSTM1/LC3B, SQSTM1/GL1, NIX/LC3B and NIX/GL1 specific interactions using P-LISA following autophagy flux inhibition

**Fig 4:**
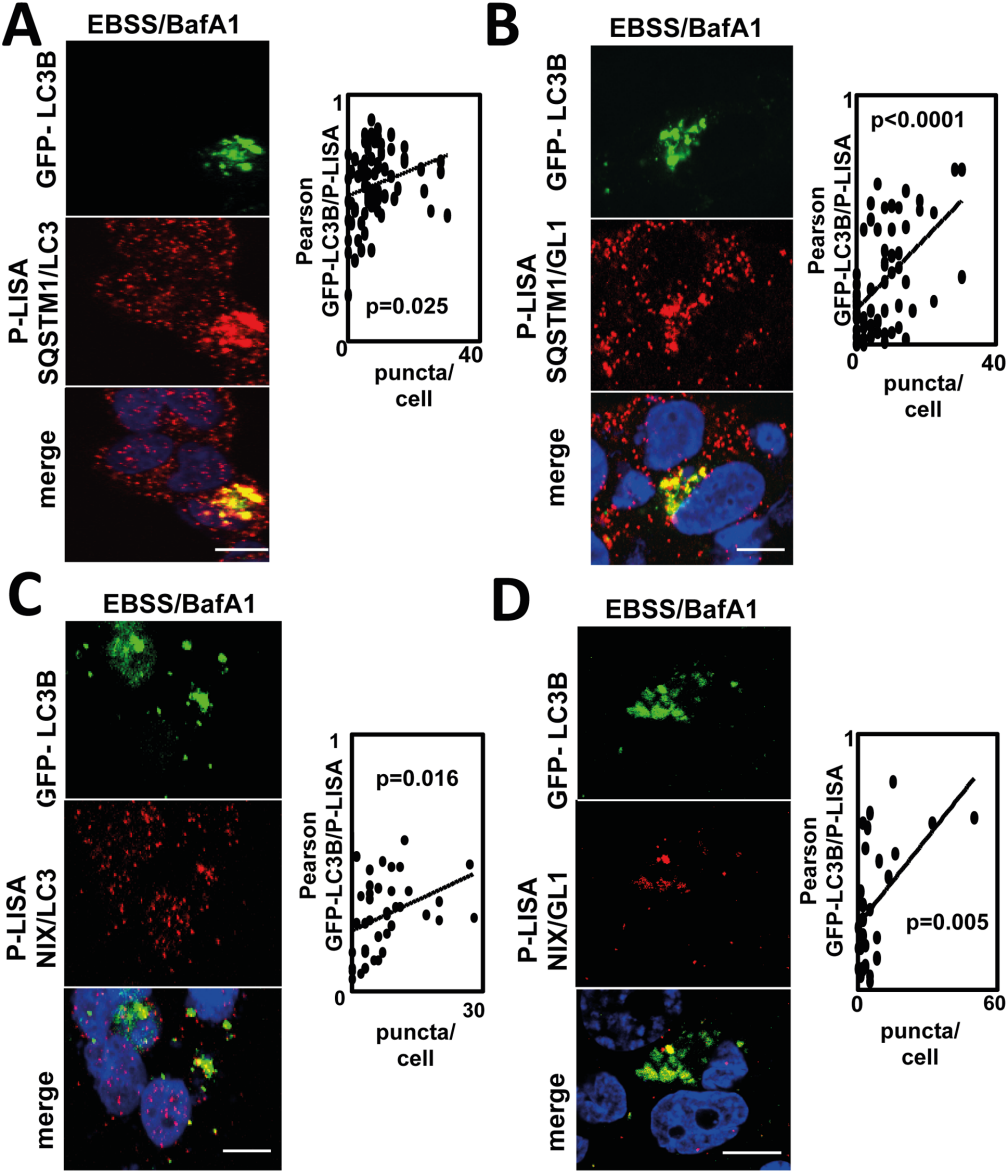
Co-localization of SQSTM1/LC3B, SQSTM1/GL1, NIX/LC3B and NIX/GL1 P-LISA signals with the GFP-LC3B protein. MDA-MB-436 cells were transfected with the pEGFP-LC3B vector then cultured with EBSS and BafA1 (100 nM) for 2 h. (**A**) Co-localization of SQSTM1/LC3B P-LISA signal and GFP-LC3B fluorescence. (**B**) Co-localization of SQSTM1/GL1 P-LISA signal and GFP-LC3B fluorescence. (**C**) Co-localization of NIX/LC3B P-LISA signal and GFP-LC3B fluorescence. (**D**) Co-localization of NIX/GL1 P-LISA signal and GFP-LC3B fluorescence. Co-localization of P-LISA signal and GFP-LC3B puncta was determined in at least 30 cells using the imageJ software. Nuclei were stained with DAPI. ****: p≤0.0001 and ***: p≤0.001, vs control (n = 3). Scale bar: 20μm.

In order to analyze whether the autophagy protein interactions could be detected following autophagy induction using P-LISA and whether they were indeed associated to the formation of autophagosomes, we asked whether the signals obtained with SQSTM1/LC3B P-LISA, SQSTM1/GL1 P-LISA, NIX/LC3B P-LISA, or NIX/GL1 P-LISA co-localized with the fusion protein GFP-LC3 overexpressed in MDA-MB-436 cells. As expected, GFP-LC3 puncta significantly colocalized with all four P-LISA signals suggesting that these signals were related to autophagosomes (Fig 4A–4D). These conclusions were confirmed by the significant correlation index determined between the number of GFP-LC3B puncta and the SQSTM1/LC3B P-LISA signals (p = 0.025) (Fig 4A). However, due to the overexpression of GFP-LC3B in transfected cells, the strong increase of SQSTM1/LC3B P-LISA signals linked to large puncta in the cells was influenced by GFP-LC3 overexpression and not only by autophagy induction since expression of GFP-LC3B significantly increased SQSTM1/LC3B P-LISA signals in both the absence or presence of EBSS/BafA1 treatment in MDA-MB-436 cells (S2 Fig). Indeed, it has already been demonstrated that the overexpression of LC3B can induce the formation of cellular aggregates which could also include SQSTM1.[15] Taken together, these observations suggest that SQSTM1/LC3B P-LISA quantification of endogenous proteins interactions may be more accurate than overexpressed GFP-LC3B quantification for autophagy flux analysis. Similar experiments performed using SQSTM1/GL1 P-LISA revealed a very strong correlation between the number of GFP-LC3B vesicles and P-LISA signals (p<0.0001) (Fig 4B). These experiments were also performed using NIX/LC3B P-LISA or NIX/GL1 P-LISA and, once more, a significant co-localization between GFP-LC3-positive puncta and P-LISA signals were observed (Fig 4C, p = 0.016 and Fig 4D, p = 0.005).

### P-LISA can be used to monitor selective autophagy

Following the demonstration that P-LISA can quantify differences in autophagosome formation, we next asked whether this technique might also help discriminating between responses to different autophagy inducers. Indeed, until now, none of the current techniques used to quantify autophagy may specifically and undoubtedly discriminate between non selective and selective autophagy. To do so, we decided to use P-LISA protocols including cargo adapters (such as NIX or SQSTM1) and ATG8 proteins (LC3B or GABARAPL1) since these proteins have been described to play an essential role in autophagy selectivity.[2,5]

**Fig 5:**
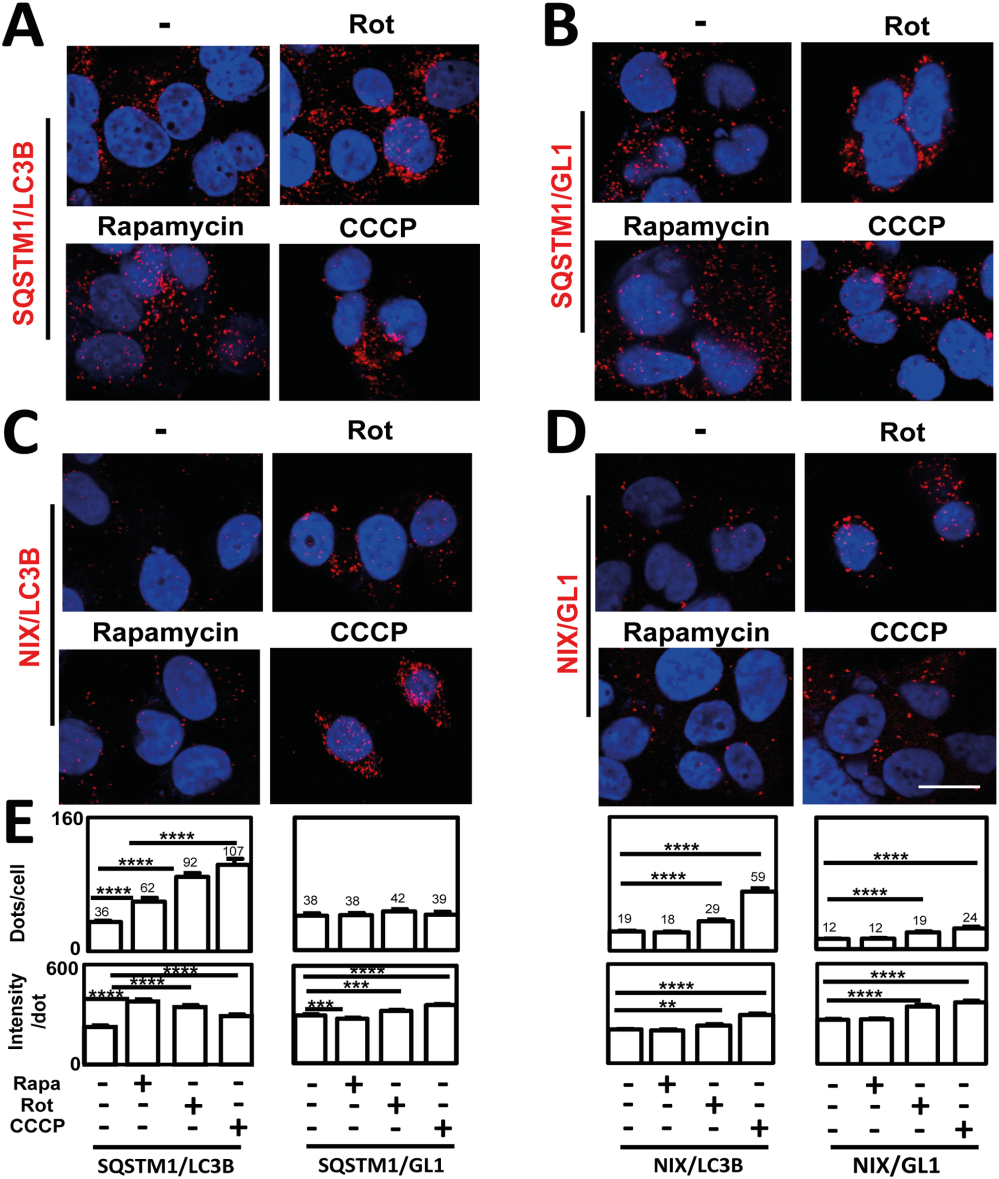
Effects of Rapamycin, Rotenone and CCCP on ATG8/cargo adapter interactions. MDA-MB436 cells were cultured for 24 h at 37°C and 5% CO_2_ then with Rapa (10 μM for 5h), Rot (50 μM for 24h) or CCCP (100 μM for 24h). (**A**) SQSTM1/LC3B, (**B**) SQSTM1/GL1, (**C**) NIX/LC3B and (**D**) NIX/GL1 P-LISA were performed according to the manufacturer’s recommendations using mouse anti-NIX or anti-SQSTM1 and rabbit anti-GL1 or anti-LC3B antibodies. Nuclei were stained with DAPI. Each picture is representative of a typical cell staining observed in 10 fields chosen at random. (**E**) Quantification of P-LISA signals (dots/cell and intensity per dot) was performed using the Blobfinder software. Each bar (Mean ± SEM) represents the mean obtained from the quantification of signals observed in about 200 cells chosen randomly in 5 different fields from 3 independent experiments. ****: p≤0.0001, ***: p≤0.0001, and **: p≤0.001, vs control. Scale bar: 20μm.

In this assay, we treated MDA-MB-436 cells, described to express endogenous SQSTM1, LC3B, NIX and GL1, with different modulators of autophagy: i) rapamycin (Rapa), an autophagy inducer inhibiting mTOR activity; ii) rotenone (Rot), an inhibitor of oxidative phosphorylation complex I or, iii) CCCP (carbonyl cyanide m-chloro-phenyl hydrazone), an uncoupling agent. The two latter chemicals have been described to induce a mitochondrial stress and mitophagy.[23,24,25] As expected, using western-blotting, an increase of the autophagosome-linked LC3B form (LC3B-II) was observed following treatment with Rapa, BafA1, CQ, Rot and CCCP but we were unable to detect any significant differences in autophagy induction following these treatments using this technique (S3 Fig). To determine whether SQSTM1/LC3B, SQSTM1/GL1, NIX/LC3B and NIX/GL1 interactions are differentially modulated by these treatments, we quantified both the number of P-LISA signals as well as the intensity per dot in untreated and treated MDA-MB-436 cells (Fig 5). Since autophagic vesicles frequently accumulate in clusters, the result is a partial or total overlap of the P-LISA signals. This explains why close interactions could not be independently quantified. However, this concentration of signals in the same area of the cell led to the increase of intensity per dot and this parameter is therefore useful to quantify autophagy since a strong increase of intensity per dot, without an apparent increase of dots per cell, also corresponds to a significant increase in ATG/cargo adapter interactions which are preferentially concentrated in specific punctates.

All treatments significantly increased SQSTM1/LC3B P-LISA signals (Fig 5A and 5E) suggesting an accumulation of autophagosomes in the cells. Moreover, SQSTM1/LC3B PLISA intensity per dot was also increased following exposure to the three autophagy inducers suggesting a relocalization of SQSTM1/LC3B interactions in these vesicles. Although the increase of intensity per dot is quite similar (about 1.5 to 2 fold) following treatment with the different autophagy inducers, the highest increase in intensity per dot and the lowest increase in dots per cell was obtained after Rapa treatment whereas the lowest increase in intensity per dot and the highest increase in dots per cell was induced by CCCP. These results suggest that these three autophagy inducers increased SQSTM1/LC3B interactions but that SQSTM1/LC3B interactions are more concentrated in specific areas of the cells following Rapa treatment.

CCCP and Rot treatments led to the highest increase in SQSTM1/LC3B P-LISA signals. As expected, transfection of *LC3B* siRNA extinguished SQSTM1/LC3B P-LISA signals in cells treated with CCCP demonstrating, once again, the specificity of these signals (S4 Fig). On the opposite, no variations in the number of SQSTM1/GL1 P-LISA signals were observed following the different treatments (Fig 5B and 5E). Interestingly, when we analyzed the interactions involving NIX, an autophagy adaptor described to be involved in mitophagy, we only observed an increase in P-LISA signals following treatments using specific mitochondrial stress inducers (Fig 5C, 5D and 5E).

Altogether, our P-LISA experiments led to the conclusion that NIX/GL1 and LC3B/NIX P-LISA can be used to specifically monitor mitophagy while SQSTM1/LC3B P-LISA, but not SQSTM1/GL1 P-LISA, is more suitable to study overall autophagy levels.

## Discussion

Whereas autophagy quantification is a crucial point when studying autophagy or biological pathways regulating autophagy, end-points analysis remain often difficult to interpret due to the dynamic autophagy flux which corresponds to the sum of initiation and degradation. Several methods have been developed by the “autophagy community” to quantify autophagy but western-blotting of SQSTM1 and LC3B-II moieties still remains the most popular experiment to estimate autophagy.[12] However, some recent data showing the frequent transcriptional up-regulation of *SQSTM1* following different autophagy-inducing stresses have demonstrated that the levels of this protein cannot exclusively be linked to autophagy degradation anymore.[26]

Quantification of fluorescent vesicles following pGFP-LC3B or pmRFP-LC3B expression is also considered as a powerful tool useful for autophagy quantification but is limited to cells in culture. Moreover, fluorescence intensities of the GFP or mRFP reporter proteins are difficult to quantify due to variable levels of expression in transfected cells. In addition, some cells remain difficult to transfect and autophagy flux could not be analyzed using this protocol. Moreover, it has been shown that overexpression of GFP-LC3B can induce the formation of aggregates independent of its role in autophagy.[15,27] More recently, the development of a new vector called ptf-LC3B leading to the expression of a double-tagged GFP-RFP-LC3B protein has helped in the study of autophagy flux. Indeed, this recombinant protein allows to distinguish between autophagosomes and autophagolysosomes. Nevertheless, the quantification of red and yellow dots still remains difficult to perform in cells and this protocol is still linked to the necessity of high transfection rates. Moreover, co-localization of this double fluorescent protein with other ATG proteins remains almost impossible to interpret. These data strongly suggest that autophagy should be analyzed by a combination of protocols and techniques.

Recent new data have also demonstrated that autophagy can be non selective or selective and that these two processes might require different autophagy proteins in the cells. For example, some specific ATGs or autophagy-related proteins, such as NIX, have been described to be specifically involved in selective autophagy of mitochondria, called mitophagy. Therefore, in order to improve the quantification of autophagy and to better discriminate between non selective or selective autophagy, we developed a P-LISA protocol to specifically monitor ATG8/cargo adapter interactions. First we asked whether this technique could be used to quantify cellular autophagy levels and, then, to discriminate between different types of induced autophagy. Indeed, the currently available techniques are preferentially used to quantify autophagic flux (WB, IF) and are not always sensitive enough to quantify slight modifications of autophagy levels. Our data confirmed that the SQSTM1/LC3B and SQTSM1/GL1 P-LISA signals detected in the cells were indeed specifically related to autophagy as demonstrated by their strong co-localization with GFP-LC3B vesicles in autophagy-induced cells following an EBSS/BafA1 treatment. Then, our data led to the conclusion that SQSTM1/LC3B interactions measured by P-LISA were increased in all treatments described to induce autophagy and might be used to quantify overall autophagy rates. On the opposite, NIX/GL1 and NIX/LC3B P-LISA appeared to be useful to discriminate between rapa-induced autophagy and specific degradation of mitochondria and therefore might be used to quantify selective mitophagy.

In conclusion, our work proves that P-LISA can be used as a new tool for autophagy quantification and for the discrimination between different induced selective autophagy. Moreover, since this technique can be adapted to the study of protein interactions in parrafine-included tissue samples or *in vivo* biological tissues, this technique could become an useful tool to perform diagnostic test targeting endogenous autophagy proteins.[28]

## Supporting Information

S1 Fig Schematic representation of the P-LISA protocol. (A) Two primary antibodies issued from distinct species recognize specific endogenous ATG proteins supposed to interact. (B) Secondary antibodies called Proximity Ligation Assay (PLA) probes, each coupled to an oligonucleotide + or an oligonucleotide—, recognize the two primary antibodies. (C) A circular probe targets the PLA + and—, only if the distance between these probes is less than 40nm. (D) Circular DNA is amplified by a DNA polymerase. (E) DNA probes coupled to fluorochromes hybridize to amplicons and lead to the formation of intracellular fluorescent dots specific to the interaction of the two ATG proteins.
(TIF)

S2 Fig Effect of EBSS/BafA1 treatment and influence of GFP-LC3B expression on SQSTM1/LC3B P-LISA signals. MDA-MB-436 cells were transfected with the pEGFP-LC3B vector then treated or not with EBSS and BafA1 (100 nM) for 2 h (left panel). SQSTM1/LC3B P-LISA signals were quantified in about 20 cells (right panel). Nuclei were stained with DAPI. ****: p≤0.0001 and **: p≤0.01.
(TIF)

S3 Fig Effects of Rapamycin, BafA1, CQ, Rotenone and CCCP on LC3B-II levels. MDA-MB436 cells were cultured for 24 h at 37°C and 5% CO_2_ then treated with Rapa (10 μM for 5h, BafA1 (100 nM for 2h), Chloroquin (40 μM for 2h)), Rot (50 μM for 24h), or CCCP (100 μM for 24h). Accumulation of the LC3B-II form was determined by western blotting using an anti-LC3B antibody.
(TIF)

S4 Fig SQSTM1/LC3B P-LISA in cells transfected with *siLC3B* and treated with CCCP. MDA-MB-436 cells were cultured for 24 h at 37°C and 5% CO_2_ and treated with CCCP (100 μM for 24h). P-LISA was performed according to the manufacturer’s recommendations. Inhibition of SQSTM1/LC3B P-LISA signals was observed in cells previously transfected with *siLC3B* compared to cells transfected with a *siRNA* control, Nuclei were stained with DAPI. Each picture is a representative image of a typical cell staining observed in 10 fields chosen at random.
(TIF)
