# Supplementary figures and images for: Proximity Ligation In situ Assay is a Powerful Tool to Monitor Specific ATG Protein Interactions following Autophagy Induction
Source: PLoS One. 2015 Jun 2;10(6):e0128701. doi: 10.1371/journal.pone.0128701 (PMC4452782; doi:10.1371/journal.pone.0128701)

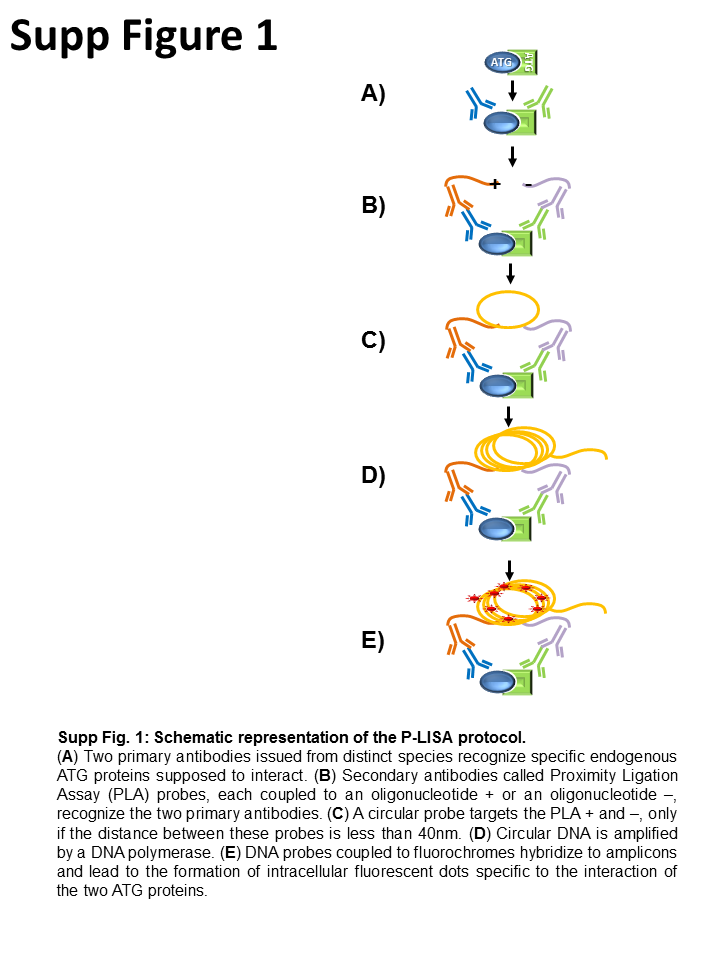

Supplement: S1 Fig — (A) Two primary antibodies issued from distinct species recognize specific endogenous ATG proteins supposed to interact. (B) Secondary antibodies called Proximity Ligation Assay (PLA) probes, each coupled to an oligonucleotide + or an oligonucleotide—, recognize the two primary antibodies. (C) A circular probe targets the PLA + and—, only if the distance between these probes is less than 40nm. (D) Circular DNA is amplified by a DNA polymerase. (E) DNA probes coupled to fluorochromes hybridize to amplicons and lead to the formation of intracellular fluorescent dots specific to the interaction of the two ATG proteins. (TIF) [file pone.0128701.s001.TIF]

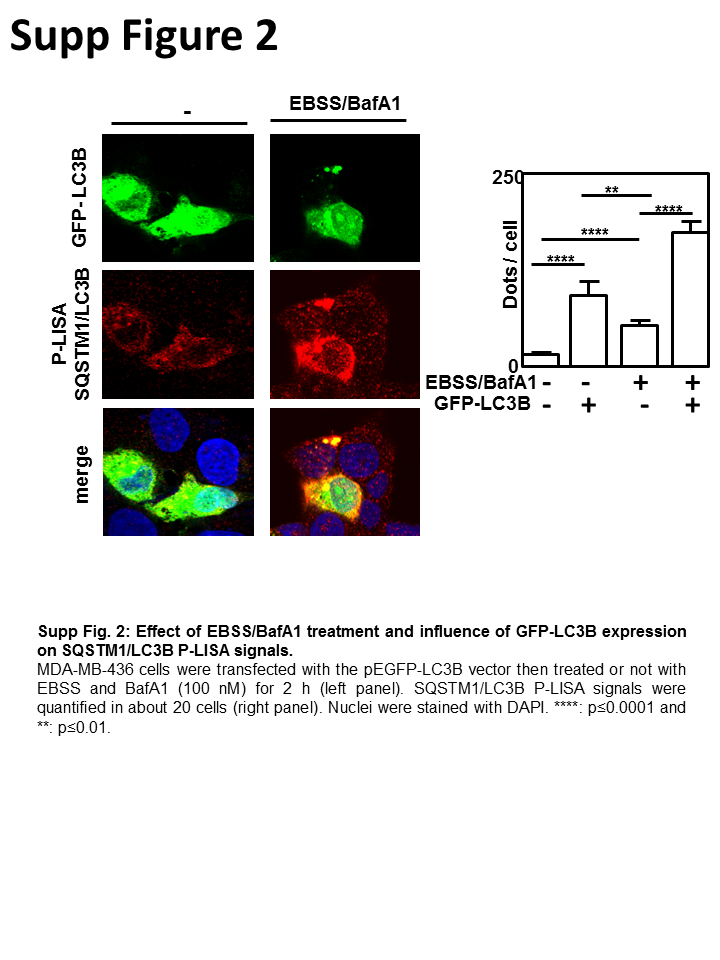

Supplement: S2 Fig — MDA-MB-436 cells were transfected with the pEGFP-LC3B vector then treated or not with EBSS and BafA1 (100 nM) for 2 h (left panel). SQSTM1/LC3B P-LISA signals were quantified in about 20 cells (right panel). Nuclei were stained with DAPI. ****: p≤0.0001 and **: p≤0.01. (TIF) [file pone.0128701.s002.TIF]

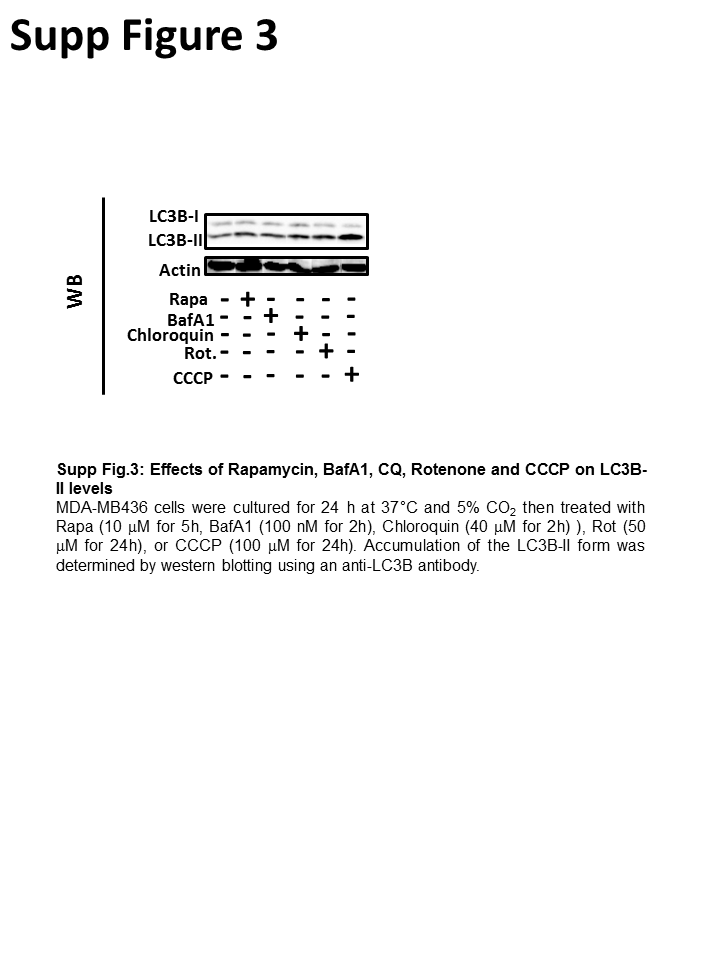

Supplement: S3 Fig — MDA-MB436 cells were cultured for 24 h at 37°C and 5% CO2 then treated with Rapa (10 μM for 5h, BafA1 (100 nM for 2h), Chloroquin (40 μM for 2h)), Rot (50 μM for 24h), or CCCP (100 μM for 24h). Accumulation of the LC3B-II form was determined by western blotting using an anti-LC3B antibody. (TIF) [file pone.0128701.s003.TIF]

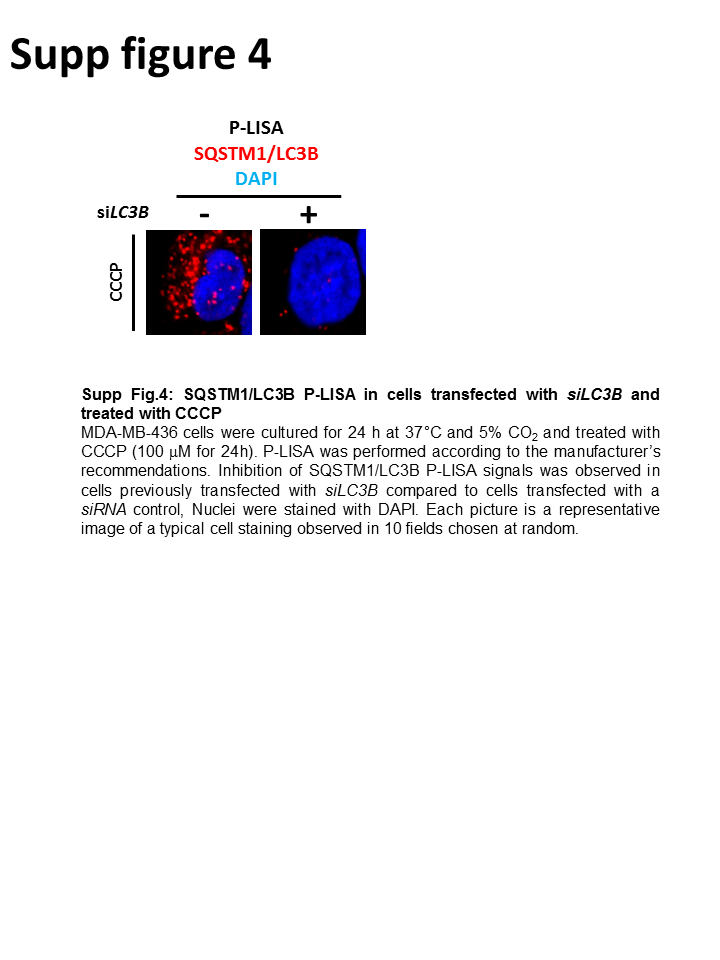

Supplement: S4 Fig — MDA-MB-436 cells were cultured for 24 h at 37°C and 5% CO2 and treated with CCCP (100 μM for 24h). P-LISA was performed according to the manufacturer’s recommendations. Inhibition of SQSTM1/LC3B P-LISA signals was observed in cells previously transfected with siLC3B compared to cells transfected with a siRNA control, Nuclei were stained with DAPI. Each picture is a representative image of a typical cell staining observed in 10 fields chosen at random. (TIF) [file pone.0128701.s004.TIF]
